# Mental and behavioral disorders related to work in Brazil: temporal trends and the impact of the Social Security Technical Nexus

**DOI:** 10.1590/0102-311XEN031524

**Published:** 2024-09-23

**Authors:** Claudio José dos Santos, Frida Marina Fischer

**Affiliations:** 1 Faculdade de Saúde Pública, Universidade de São Paulo, São Paulo, Brasil.

**Keywords:** Occupational Accidents, Mental Disorders, Social Security, Occupational Health, Time Series Studies, Acidentes de Trabalho, Transtornos Mentais, Previdência Social, Saúde Ocupacional, Estudos de Séries Temporais, Accidentes de Trabajo, Trastornos Mentales, Seguridad Social, Salud Laboral, Estudios de Series Temporales

## Abstract

This article aimed to assess the temporal trend of work-related mental and behavioral disorders in Brazil, as well as to measure the effect of changes in the implementation of the Social Security Technical Nexus (NTP, acronym in Portuguese) on the incidence of these disorders among beneficiaries of the General Social Security System (RGPS, acronym in Portuguese). It is an analysis of time series and interrupted time series with data from the Brazilian Ministry of Social Security information system on cases of work-related mental and behavioral disorders from 2003 to 2019. The Prais-Winsten method was employed to calculate the annual percent change (APC) of the incidence rates for the conditions under study. The average incidence of work-related mental and behavioral disorders was 35.48 per 100,000 RGPS links during the period 2003-2019, with an increasing trend (APC = 9.67%; p = 0.033) for Brazil. Before the implementation of changes in the NTP (2003-2007), this value was 15.59, with an increasing trend (APC = 29.28%; p < 0.001), and it more than doubled (43.77) after the RGPS modified the way of establishing the nexus between illness and work (2008-2019). The post-NTP trend for work-related mental and behavioral disorders was a decrease in the country (APC = -23.73%; p < 0.001), a pattern that was repeated for all regions of the country. The findings suggest that the changes in the way of establishing the NTP between illness and work represented an advancement in the system of recording and notifying work-related mental and behavioral disorders in Brazil.

## Resumo

Este artigo teve como objetivo avaliar a tendência temporal dos transtornos mentais e comportamentais relacionados ao trabalho no Brasil, bem como medir o efeito das mudanças na implementação do Nexo Técnico Previdenciário (NTP) sobre a incidência desses transtornos entre os beneficiários do Regime Geral de Previdência Social (RGPS). Trata-se de um estudo de séries temporais e séries temporais interrompidas que utilizou dados do sistema de informações do Ministério da Previdência Social sobre casos de transtornos mentais e comportamentais relacionados ao trabalho de 2003 a 2019. O método de Prais-Winsten foi empregado para calcular a variação percentual anual (VPA) das taxas de incidência para as condições em estudo. A incidência média de transtornos mentais e comportamentais relacionados ao trabalho foi de 35,48 por 100.000 vínculos do RGPS durante o período de 2003 a 2019, com uma tendência de aumento (VPA = 9,67%; p = 0,033) para o Brasil. Antes da implementação das mudanças no NTP (2003-2007), esse valor era de 15,59, com uma tendência de aumento (VPA = 29,28%; p < 0,001), e mais que dobrou (43,77) depois que o RGPS modificou a forma de estabelecer o nexo entre doença e trabalho (2008-2019). A tendência pós-NTP para transtornos mentais e comportamentais relacionados ao trabalho foi uma diminuição no país (VPA = -23,73%; p < 0,001), um padrão que se repetiu para todas as regiões do país. Os resultados sugerem que as mudanças na forma de estabelecer o NTP entre doença e trabalho representaram um avanço no sistema de registro e notificação de transtornos mentais e comportamentais relacionados ao trabalho no Brasil.

## Resumen

Este artículo tuvo como objetivo evaluar la tendencia temporal de los trastornos mentales y de conducta relacionados con el trabajo en Brasil, así como medir el efecto de los cambios en la implementación del Nexo Técnico de la Seguridad Social (NTP, por sus siglas en portugués) sobre la incidencia de estos trastornos entre los beneficiarios del Sistema General de Seguridad Social (RGPS, por sus siglas en portugués). Se trata de un estudio de series temporales y series temporales interrumpidas que utilizó datos del Ministerio de Seguridad Social sobre casos de trastornos mentales y de conducta relacionados con el trabajo de 2003 a 2019. Se utilizó el método Prais-Winsten para calcular el cambio porcentual anual (CPA) de las tasas de incidencia para las afecciones en estudio. La incidencia promedio de trastornos mentales y de conducta relacionados con el trabajo fue de 35,48 por 100.000 vínculos del RGPS durante el período 2003 a 2019, con una tendencia al alza para Brasil (CPA = 9,67%; p = 0,033). Antes de implementar los cambios en la NTP (2003-2007), este valor era de 15,59, con una tendencia al alza (CPA = 29,28%; p < 0,001), y se duplicó (43,77) después de que el RGPS modificara la forma de establecer el vínculo entre enfermedad y trabajo (2008-2019). La tendencia post-NTP para trastornos mentales y de conducta relacionados con el trabajo fue una disminución en el país (CPA = -23,73%; p < 0,001), patrón que se repitió para todas las regiones del país. Los resultados revelan que los cambios en el modo de establecer el NTP entre la enfermedad y el trabajo representaron un avance en el sistema de registro y notificación de trastornos mentales y de conducta relacionados con el trabajo en Brasil.

## Introduction

Mental and behavioral disorders often emerge among cases of illness, frequently proving incapacitating. These conditions are among the leading causes of sickness-related absenteeism, leading to decreased productivity due to lost workdays ^1^ ^,^ ^2^ ^,^ ^3^. In 2019, mental illness was the third main cause of disability benefit grants by the Brazilian Social Security system, accounting for 9% of the total, with 5% of the cases being work-related ^4^.

The recognition of these conditions as labor-related in Brazil dates back primarily to the 1990s and 2000s, when the psychological repercussions of work began to gain attention in clinics and also in academic, scientific, and governmental spheres ^5^. The debate on mental and behavioral disorders as a work-related illness was included in the political health agenda specifically focused on workers during the 2nd National Conference on Workers’ Health, held in 1994 ^6^. These disorders, which were not previously recognized as occupational accidents, exposed the complexity of assessing the relation between the health/disease process and work.

Work-related mental and behavioral disorders do not arise from isolated factors, but result from the interaction between the work environment, the body, and the psychological apparatus of workers ^7^. The influence of work on mental health covers various aspects, ranging from specific exposures to chemical agents, such as pesticides and heavy metals, to the complex combination of organizational factors, such as workload, imbalance in responsibilities and power, personnel management policies, and the hierarchical structure within organizations ^8^ ^,^ ^9^ ^,^ ^10^ ^,^ ^11^.

Work-related mental and behavioral disorders, therefore, are conditions with a complex and multifactorial origin, encompassing biological and genetic aspects, social factors, as well as access to care and the development of resilience, such as education and social support. For this reason, establishing the so-called causal nexus between an mental and behavioral disorders and work is often challenging.

The implementation of the Social Security Technical Nexus (NTP, acronym in Portuguese) introduced a new approach to analyzing work disability by the medical experts of the Brazilian National Social Security Institute (INSS, acronym in Portuguese). This system, operative since its adoption in April 2007, allows for the identification of the relation between injury and work activity by comprehensively analyzing the conditions that lead to the disability, whether clinical or subclinical in nature ^12^ ^,^ ^13^.

Since April 2007, therefore, establishment of the nexus between injuries and work within the Social Security system has been modified, implementing three key measures: the automation of Lists A and B of Annex II of *Decree n. 3,048/1999*, which had already been operating since 1999 but were previously underused in practice during medical-expert examinations. These lists, containing more than 200 work-related illness, were integrated into the Benefit Incapacity Evaluation System (SABI, acronym in Portuguese), used by INSS experts for the preparation of medical-expert reports. In addition, the requirement for the Communication of Occupational Accident (CAT, acronym in Portuguese) during medical examination, as a prerequisite for establishing the nexus, was waived, and the Technical Epidemiological Social Security Nexus (NTEP, acronym in Portuguese) was implemented, resulting in the creation of List C of Annex II of *Decree n. 3,048/1999*, also automated in SABI ^14^ ^,^ ^15^.

The three mentioned measures encompassed the creation of a new methodology for granting accident benefits and marked the implementation of the NTP, subdivided into three types:

(1) Professional or Work Technical Nexus is established when there is an association of the injury with the etiological agents or risk factors present in the employers economic activities, listed in Lists A and B of Annex II of the Social Security Regulations.

(2) Individual Technical Nexus for typical work accidents or during commutes, as well as in special conditions of work or directly related to it.

(3) NTEP is applied considering the statistical significance of the association between the International Statistical Classification of Diseases and Related Health Problems - 10th revision (ICD-10) and the Brazilian National Classification of Economic Activities (CNAE, acronym in Portuguese), as established in List C in Annex II of the Social Security Regulations ^14^.

The occurrence of any of the three nexuses results in the recognition of the condition as work-related and the granting of an accident benefit, while the absence of them classifies the condition as not work-related and the benefit as social security ^14^ ^,^ ^15^. With this approach, it a CAT no longer mandatory to characterize a disability as accident-related ^14^ ^,^ ^15^.

The introduction of changes in the recognition of the nexus between injury and work by the INSS caused a shift in how Social Security analyzes cases of work disability, as well as in the establishment of causal nexus in cases of work injuries.

Despite this consideration, there is a lack of recent research dedicated to evaluating trends in work-related mental and behavioral disorders in the Brazilian context, as well as a scarcity of studies aimed at measuring the impact of NTP on the incidence of work-related mental and behavioral disorders in the country. The few identified initiatives have a predominantly descriptive nature ^16^ ^,^ ^17^. There is also a shortage of time-series studies that have analyzed the distribution of disabilities according to ICD-10 codes (such as those in Chapter V, addressing mental and behavioral disorders), and of initiatives aimed at identifying whether these occurrences have been less frequent after the implementation of legislative changes, such as the NTP, and public policies in general. These analyses are essential for evaluating improvements in health indicators related to occupational accidents in Brazil and the impact of coping strategies ^18^.

Understanding the temporal trends of work-related mental and behavioral disorders, coupled with the analysis of the public policies effects on worker’s health and other legislative changes, such as those in establishing the NTP, is a necessary condition for the development and continuous improvement of effective strategies for protecting, promoting, and preventing mental health issues in work environments. It is also important for the mitigation of socioeconomic impacts associated with absences and disabilities due to work-related mental and behavioral disorders.

Thus, this article aimed to evaluate the temporal trend of mental and behavioral disorders in the context of occupational accidents in Brazil, as well as to measure the effect of changes in the establishment of the NTP on these occurrences incidence among beneficiaries of the General Social Security System (RGPS, acronym in Portuguese).

## Materials and methods

This study employed a time series and interrupted time series design to analyze the incidence rates of work-related mental and behavioral disorders in Brazil.

On January 14, 2024, we collected data from the Historical Database of Occupational Accidents (AEAT InfoLogo; https://www3.dataprev.gov.br/aeat/inicio.htm), an information system of the Brazilian Ministry of Social Security that provides aggregated information on the numbers of occupational accidents and insured workers under the RGPS. AEAT is a publication that presents comprehensive data on occupational accidents, including their consequences, affected economic sectors, and geographic location of events. The AEAT InfoLogo database allows retrieving information published in all editions of AEAT, enabling the creation of customized tables and graphics.

The annual quantities of work-related mental and behavioral disorders (ICD-10 F00-F99, Chapter V) were extracted between January 2003 and December 2019. The year 2003 was selected due to data availability in the source, and the decision to end the analysis in 2019 was motivated by avoiding possible distortions from the effects of the global pandemic caused by the COVID-19 on the outcome of interest. These data were tabulated for Brazil, by administrative region (North, Northeast, Southeast, South, and Central-West), and according to ICD-10 codes. The annual incidence rate for these conditions was calculated using the equation: incidence rate = (number of new occupational accidents by ICD-10 F00-F99 / average number of work links) × 100,000.

To calculate the annual percent change (APC) of the temporal trends in the incidence rate of work-related mental and behavioral disorders for the country, by administrative region and according to ICD-10 codes, the Prais-Winsten method was used. This method allows for the correction of first-order autocorrelation of error terms. The dependent variable was the base-10 logarithm of the rates, and the independent variable was the years of the time series.

For calculating the APC and 95% confidence intervals (95%CI), the formulas recommended by Antunes & Waldman ^19^ were used in order: APC = -1+10^b^; 95%CI = -1+10^b±t×SE^.

In the formulas, “b” and the standard error (SE) values were obtained by regression analysis. The “t” value is obtained from the Student’s t-distribution table. Based on this procedure, the trend was classified as increasing, decreasing, or stationary. The trend was considered stationary when the coefficient of the regression equation for this parameter was equal to zero in the hypothesis test (p ≥ 0.05).

To assess the impact of NTP, the historical series of work-related mental and behavioral disorders incidence in RGPS beneficiaries for the country and by macroregion were divided into two segments for periods: before and after NTP. The division aimed to quantify both immediate (level) and trend (slope) changes.

In this study, the post-intervention period started in 2008, which is the first full year of NTP’s effectiveness in Brazil. The previous years, including 2007 when NTP was implemented in April 2007, were considered pre-intervention.

The regression equation Y_i_ = b_0_+b_1_×time+b_2_×level+b_3_×trend was used in this model. Here, b_1_ represents the trend measure of the period preceding the implementation of the NTP; b_2_ is the level change, i.e., the immediate impact of the NTP; and b_3_ is the trend change, representing the period after the NTP implementation ^20^. Additionally, the means of the rates for pre- and post-intervention periods were calculated.

For the series graphical presentation, historical series graphs were created for work-related mental and behavioral disorders incidence values before and after the implementation of NTP. The graphs were made using Microsoft Excel 2016 (https://products.office.com/), and trend analysis was performed using Stata, version 17.1 (https://www.stata.com).

### Ethical aspects

This research exclusively used publicly available data from an official source, preventing subject identification, and was analyzed in an aggregated manner. For these reasons, the project was not submitted to a research ethics committee, as stipulated by *Resolution n. 510/2016* (Brazilian National Health Council).

## Results

In Brazil, a total of 239,951 work-related incapacities due to work-related mental and behavioral disorders were registered by the RGPS between the years 2003 and 2019. Out of these records, 66.2% (n = 158,847) corresponded to neurasthenic, stress-related, and somatoform disorders (F40-F48), followed by 28.4% (n = 68,157) that were attributed to mood disorders (F30-F39). The remaining conditions, represented by ICD-10 codes F00-F09, F10-F19, F20-F29, F50-F59, F60-F69, F70-F79, F80-F89, and F90-F99, accounted for 5.4% of the total notifications of work-related mental and behavioral disorders.

The average incidence (μ) of work-related mental and behavioral disorders was 35.48 per 100,000 RGPS links during the period 2003-2019, with a significant increasing trend (APC = 9.67%; p = 0.033). This upward trend was also observed in the North (APC = 14.06%; p = 0.002), Northeast (APC = 13.52%; p = 0.020), and Southeast (APC = 9.65%; p = 0.027) regions. The South and Central-West regions showed a stationary trend (Table 1).

**Table 1:** Time series and temporal trend of the incidence (per 100,000 links) of work-related mental and behavioral disorders among beneficiaries of the Social Security in Brazil, 2003-2019, according macroregions.

Year	Brazil	North	Northeast	Southeast	South	Central-West
2003	8.30	5.27	4.91	8.31	11.16	10.43
2004	11.18	6.01	7.01	13.84	9.57	7.25
2005	14.96	7.13	7.30	19.36	11.82	11.62
2006	12.69	6.67	6.86	14.58	14.66	10.62
2007	30.83	21.22	18.21	35.28	36.09	19.72
2008	47.39	29.88	30.27	51.61	56.72	44.18
2009	51.57	35.59	43.79	53.90	58.63	46.12
2010	42.81	31.63	40.35	46.28	43.73	29.69
2011	41.21	29.18	42.78	45.5	35.65	29.52
2012	41.09	24.43	44.02	45.98	34.61	27.93
2013	45.16	31.85	47.34	48.80	41.51	34.08
2014	43.57	32.31	40.54	48.15	42.68	30.31
2015	44.66	40.85	42.62	47.81	43.99	33.66
2016	46.55	48.42	41.96	48.84	50.69	32.68
2017	42.60	47.22	40.08	46.45	39.95	27.86
2018	40.73	50.10	39.43	43.73	37.02	28.16
2019	37.94	41.25	37.22	40.71	33.89	33.15
μ	35.48	28.77	31.45	38.77	35.43	26.88
APC (%)	9.67	14.06	13.52	9.65	7.39	7.56
95%CI	0.84; 19.27	5.88; 22.87	2.33; 25.95	1.18; 18.82	-1.83; 17.47	-0.08; 15.77
p-value	0.033	0.002	0.02	0.027	0.111	0.052
Trend	Increasing	Increasing	Increasing	Increasing	Stationary	Stationary

95%CI: 95% confidence interval; μ: mean; APC: annual percent change.

The increasing trend observed in the incidence rate of work-related mental and behavioral disorders was predominantly attributed to the categories of ICD-10 F40-F48 (APC = 7.293%; p = 0.008) and F60-F69 (APC = 6.95%; p < 0.001). Other categories, such as F00-F09, F10-F19, F20-F29, F30-F39, F50-F59, F70-F79, F80-F89, and F90-F99 did not show statistically significant trends using the employed method (Table 2).

**Table 2:** Time series and temporal trend of the incidence (per 100,000 links) of work-related mental and behavioral disorders among beneficiaries of the Social Security in Brazil, 2003-2019, according to ICD-10 codes (Chapter V).

Year	F00-F09	F10-F19	F20-F29	F30-F39	F40-F48	F50-F59	F60-F69	F70-F79	F80-F89	F90-F99
2003	0.43	0.25	0.88	6.09	0.06	0.17	0.01	0.04	0.07	0.43
2004	0.41	0.19	1.21	8.87	0.05	0.11	0.02	0.02	0.06	0.41
2005	0.27	0.17	1.63	12.25	0.06	0.15	0.04	0.05	0.07	0.27
2006	0.15	0.17	1.45	10.41	0.04	0.10	0.02	0.01	0.06	0.15
2007	0.79	0.37	11.12	17.47	0.05	0.14	0.02	0.05	0.09	0.79
2008	1.79	1.06	18.13	25.30	0.04	0.18	0.02	0.04	0.16	1.79
2009	1.82	1.21	20.06	27.55	0.09	0.13	0.02	0.03	0.13	1.82
2010	1.53	1.03	15.82	23.80	0.05	0.10	0.01	0.03	0.08	1.53
2011	1.29	0.78	14.27	24.20	0.07	0.11	0.04	0.04	0.09	1.29
2012	1.15	0.65	12.53	26.26	0.04	0.06	0.02	0.01	0.07	1.15
2013	1.05	0.52	13.33	29.71	0.04	0.10	0.02	0.02	0.13	1.05
2014	0.95	0.50	12.19	29.34	0.05	0.07	0.01	0.03	0.10	0.95
2015	0.66	0.38	9.89	33.21	0.07	0.06	0.01	0.03	0.08	0.66
2016	0.56	0.41	9.95	35.05	0.05	0.08	0.01	0.02	0.19	0.56
2017	0.46	0.32	9.03	32.30	0.04	0.04	0.02	0.03	0.07	0.46
2018	0.48	0.34	9.76	29.71	0.04	0.05	0.02	0.04	0.02	0.48
2019	0.44	0.24	11.19	25.75	0.03	0.06	0.01	0.02	0.03	0.44
μ	0.84	0.51	10.14	23.37	0.05	0.10	0.02	0.03	0.09	0.84
APC (%)	-2.92	0.76	0.22	16.08	9.29	-2.12	-6.95	-2.11	-1.70	-3.21
95%CI	-8.33; 2.81	-11.42; 14.61	-11.61; 13.63	-1.01; 36.11	2.69; 16.30	-4.72; 0.54	-8.88; -4.98	-6.15; 2.10	-4.63; 1.32	-11.25; 5.57
p-value	0.288	0.902	0.971	0.064	0.008	0.11	0	0.297	0.246	0.436
Trend	Stationary	Stationary	Stationary	Stationary	Increasing	Stationary	Decreasing	Stationary	Stationary	Stationary

95%CI: 95% confidence interval; μ: mean; APC: annual percent change.

Note: ICD-10 codes (Chapter V): F00-F09 - Organic mental disorders, including symptomatic ones; F10-F19 - Mental and behavioral disorders due to psychoactive substance use; F20-F29 - Schizophrenia, schizotypal, and delusional disorders; F30-F39 - Mood disorders; F40-F49 - Neurotic, stress-related, and somatoform disorders; F50-F59 - Behavioral syndromes associated with physiological dysfunctions and physical factors; F60-F69 - Personality disorders and adult behavior disorders; F70-F79 - Intellectual disabilities; F80-F89 - Disorders of psychological development; F90-F99 - Behavioral and emotional disorders appearing during childhood or adolescence.

The average incidence of work-related mental and behavioral disorders was 15.59 per 100,000 links before the implementation of the NTP, with an increasing trend for Brazil (APC = 29.28%; p < 0.001). A significant positive immediate impact (level change) was evidenced in the number of disability records related to work-related mental and behavioral disorders nationwide (APC = 108.80%; p < 0.001) and for all regions of the country. The average incidence of work-related mental and behavioral disorders more than doubled in the post-implementation period of the NTP (43.77/100,000) and showed a decreasing trend throughout the historical series of this resource implementation (APC = -23.73%; p < 0.001), a pattern observed for all regions of the country (Table 3).

**Table 3:** Interrupted time series of the incidence (per 100,000 links) of work-related mental and behavioral disorders among Social Security beneficiaries in Brazil, 2003-2019, according macroregions.

	Brazil	North	Northeast	Southeast	South	Central-West
Pre-NTP						
μ	15.59	9.26	8.86	18.27	16.66	11.93
b_1_	0.11	0.13	0.09	0.12	0.12	0.07
APC (%)	29.28	33.59	23.42	30.45	32.04	18.68
95%CI	20.31; 38.93	14.24; 56.22	14.36; 33.20	21.47; 40.09	12.86; 54.48	5.16; 33.94
p-value	0.000	0.002	0.000	0.000	0.002	0.009
Trend	Increasing	Increasing	Increasing	Increasing	Increasing	Increasing
Immediate impact of NTP						
b_2_	0.32	0.27	0.56	0.28	0.28	0.39
APC (%)	108.80	86.15	261.48	89.32	90.71	144.36
95%CI	67.71; 159.95	14.08; 203.77	187.64; 354.29	21.47; 40.09	17.06; 210.68	67.74; 255.98
p-value	0.000	0.017	0.000	0.002	0.013	0.000
Trend	Increasing	Increasing	Increasing	Increasing	Increasing	Increasing
Post-NTP (2008-2019)						
μ	43.77	36.89	40.87	47.31	43.26	33.11
b_3_	-0.118	-0.105	-0.093	-0.122	-0.132	-0.086
APC (%)	-23.78	-21.51	-19.21	-24.49	-26.25	-17.88
95%CI	-29.13; -18.03	-33.25; -7.71	-25.17; -12.76	-29.73; -18.85	-37.64; -12.77	-27.45; -7.06
p-value	0.000	0.007	0.000	0.000	0.002	0.004
Trend	Decreasing	Decreasing	Decreasing	Decreasing	Decreasing	Decreasing

95%CI: 95% confidence interval; μ: mean; APC: annual percent change; b_1_: estimator of the initial trend; b_2_: estimator of the immediate impact; b_3_: estimator of the trend post-NTP; NTP: Social Security Technical Nexus.

**Figure 1:**
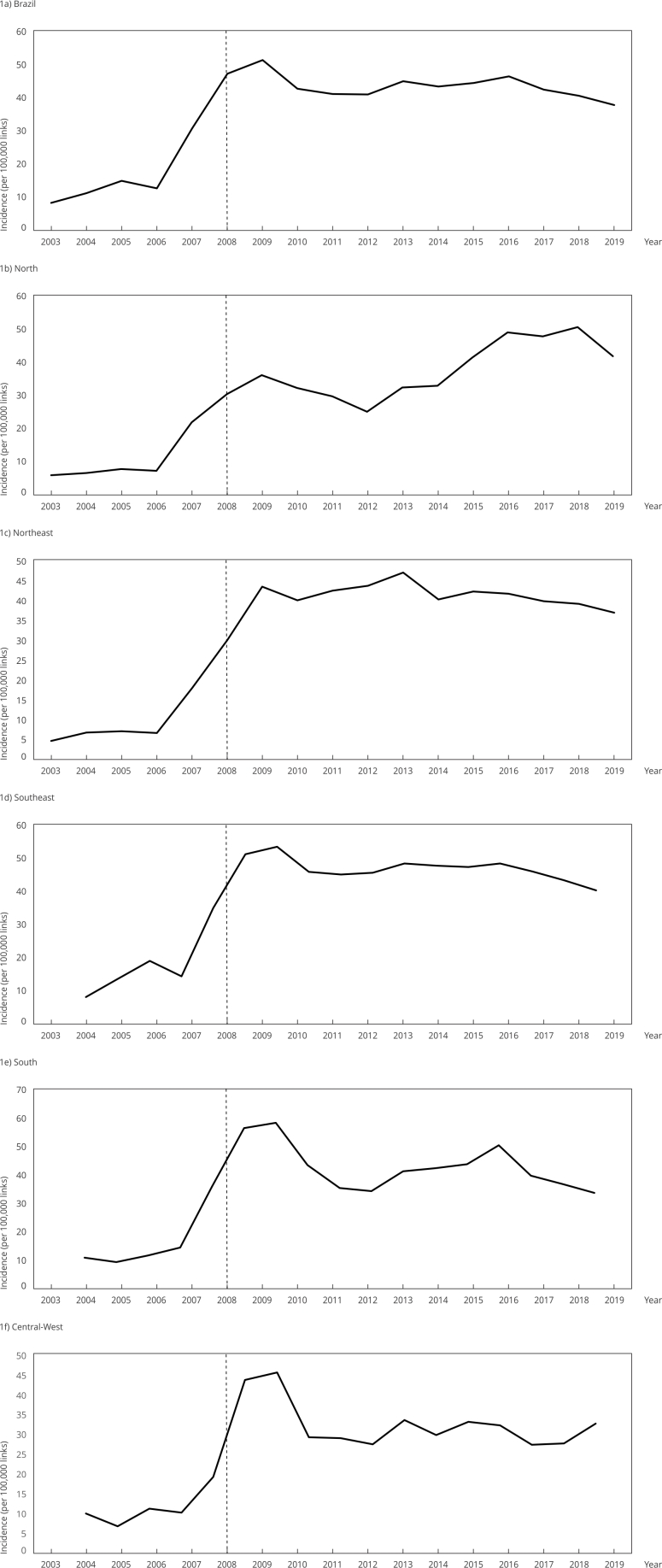
Temporal evolution of the incidence (per 100,000 links) of work-related mental and behavioral disorders among Social Security beneficiaries in Brazil, 2003-2019, according macroregions.

Figure 1 graphically summarizes the temporal evolution of the incidence of work-related mental and behavioral disorders among RGPS beneficiaries in Brazil over the seventeen years analyzed, highlighting variations in this indicator and its trend throughout the historical series.

## Discussion

This article aimed to assess the temporal trend of work-related mental and behavioral disorders in Brazil, as well as to measure the impact of changes in the establishment of the NTP, initiated in 2007. For this purpose, we used a time series analysis approach and interrupted time series, of which the latter is recognized in the literature as the most effective non-experimental approach to assess longitudinal effects of interventions and test hypotheses about factors that modify the temporal behavior of health-related measures ^21^ ^,^ ^22^ ^,^ ^23^.

The analysis of the temporal trend revealed a significant and consistent increase in the average incidence of work-related mental and behavioral disorders throughout the 2003-2019 historical series, showing a 9.65% annual increase in these conditions among RGPS contributors in the country. This rise was significant, with variations, also in the North, Northeast, and Southeast regions. Moreover, there was a stationary trend in the South and Central-West regions, which may suggest regional differences in working conditions and mental health across the country. The most significant incidence was in the ICD-10 F40-F48 categories, representing 66.2% of the total cases of work-related mental and behavioral disorders registrations, and the identification of these conditions as the main drivers of the increasing trend highlights the importance of considering them in the prevention and management of mental health in the workplace. These data are consistent with recent research that has shown a high burden and expressiveness of anxiety disorders in Brazil ^24^ ^,^ ^25^ ^,^ ^26^ and provide important insights for employers, health agencies, and other stakeholders involved in promoting worker’s health, to adopt prevention and intervention strategies.

Interrupted time series analysis indicated that the implementation of the new NTP methodology had a substantial impact on the notification and recognition of work-related mental and behavioral disorders in Brazil. Before the adoption of the NTP, the average incidence of these conditions was growing sharply, reaching 28.28% per year. The pre-NTP national average was 15.59 per 100,000 links, with regional variations between 8.86 and 18.27 per 100,000. The prevention of occupational hazards, leading to a decrease in their occurrence, would result in a reduction in the value of the Aid for Labor Incapacity Related to Work Incidence Risk Degree (GILRAT, formerly known as the Occupational Accident Insurance - SAT, acronyms in Portuguese) paid by companies to the Brazilian Ministry of Social Security, which effectively occurred in 2010 with the establishment of the Social Security Accident Factor (FAP, acronym in Portuguese). On the other hand, the rate reduction of this insurance may also have led to underreporting of hazards by companies, in exchange for minimizing the costs associated with these events ^27^, which could explain the decreasing trend observed in the historical series in the study. It is worth noting, however, that even with this decreasing trend, the national average of work-related mental and behavioral disorders remained at 43.77 per 100,000, more than double the average incidence of these hazards in the period prior to the methodology. The administrative regions of the country also presented higher incidence averages than those of the pre-NTP period, ranging from 36.89 to 47.31 per 100,000 links.

The NTEP and other measures adopted by the INSS in 2007 aimed to mitigate the underreporting of occupational accidents and work-related diseases. This methodology, in its essence, represents the acknowledgment of the limitations inherent in the old NTP, which was the exclusive basis of the classification adopted by Social Security until March 2007. This new approach includes, as workplace events, disabilities resulting from a specific economic activity that show statistical significance among various diseases and that were forwarded for the granting of social security benefits without a work-related nexus by companies ^28^, in addition to emphasizing the automation of Lists A and B. It also recognizes that the CAT does not constitute the sole source of notification of workplace accidents, as the recording of these conditions, although mandatory, is subject to the employer’s interest, which often tends toward underreporting ^29^.

In the context of the NTP, the NTEP uses descriptive epidemiology based on general prevalence studies, estimating the cumulative incidence ratio to calculate the relative risk. Its application, despite being contested by some entities and groups of researchers ^30^ ^,^ ^31^, was evaluated in a validation study, which pointed to its institutionalization as an advance in establishing the work-disease relation in the social security scope. In addition, it indicated that such a strategy expanded and diversified the diagnoses that were previously underreported ^17^. This methodology was also recognized by the Supreme Federal Court as constitutional, maintaining its validity in the country ^32^.

The findings identified reflect that the method of establishing the nexus between injury and work adopted by the INSS, in effect since 2007, of considering associations between work activity and injury; automation; and by allowing the possibility of a relation between an injury and/or accident and the company’s activity that the insured develops their activities in INSS medical examination, even in the absence of a CAT. It contributed to a significant increase in the incidence of work-related mental and behavioral disorders in Brazil and to a change in the direction of the trend of these conditions in RGPS beneficiaries, reflecting an evolution in the recognition and identification of these hazards as work-related, directly impacting benefits grants and the recognition of social security rights to workers. These findings align with empirical evidence from a study conducted by Todeschini & Codo ^33^, which pointed out that one year after the incorporation of NTP, there was a 1,578% growth in records of work leave due to conditions related to Chapter V of ICD-10. Our findings are also confirmed by other studies that show a significant increase in the frequency of work injuries in general and social security benefits in Brazil after the institution of this strategy to characterize the relation between work and illness ^16^ ^,^ ^17^ ^,^ ^30^ ^,^ ^34^ ^,^ ^35^ ^,^ ^36^ ^,^ ^37^ ^,^ ^38^.

Lombardi ^39^ argues that the utility of NTEP goes beyond the granting of social security benefits and the determination of the GILRAT. For him, NTEP is crucial for securitization, because it acts as a facilitator in demonstrating the occupational nature of the event. In addition, it is a modern approach to the theory of responsibility, allowing the presumption of the causal nexus, regardless of fault, based on the idea of inherent risk in the employer’s activity. The author argues that possible inconsistencies arising from NTEP can be rectified by appealing to the social security agency when the organization has data and information that demonstrate that the harms are not technically related to the worker’s work.

The current trend of decreasing incidence of work-related mental and behavioral disorders is an alert and points to the continuous need to improve the methods of identification and approach to these harms in the context of INSS medical examination and by the worker’s health care services, especially considering that the contemporary scenario is characterized by significant changes in employment relations and production processes, restructuring of the economy, technological advances, precariousness of labor relations, and socioeconomic pressures. In these work contexts, there is an intensification of traditional occupational exposures, as well as the emergence of exposures resulting from new demands and requirements, with an impact on the workers’ physical and mental health ^40^ ^,^ ^41^.

A significant challenge in evaluating the causal nexus of work-related mental and behavioral disorders is the neglect by social security experts regarding psychosocial risks. In a study conducted in São Paulo, it was found that among applicants for disability assistance due to common mental disorders (CMD), only 23.7% of CMD-related illnesses were officially considered work-related. The researchers criticize these results and point out the lack of standardization and/or adequate tools in INSS protocols to address psychosocial risk factors, producing a misguided evaluation of the causal nexus and the non-characterization of benefits as occupational for workers exposed to these stressors. The study they conducted showed that disability assistance requests with NTEP indication were discredited by experts, and the lack of inspection in the workplace harmed workers, excluding the causal nexus. The authors emphasize that these data may be more serious due to the underreporting of diseases to the social security agency, resulting from both intentional employer omissions and the increase in informal work ^42^. Suggestions for improving the nexus between injury and work by the INSS include incorporating workers’ job profiles, workplace inspections, continuous review of its statistical database, as well as investments in periodic training of the medical experts responsible for its application ^27^.

Another possible measure to improve its sensitivity and effectiveness would be updating its statistical base according to *Ordinance n. 1,999/2023* (Brazilian Ministry of Health) ^43^, which updated the List of Occupational Diseases (LDRT, acronym in Portuguese). This normative milestone broke with 24 years of gaps and marked a turning point in the Brazilian Workers’ Health field. It placed special emphasis on the inclusion of psychosocial factors and work-related mental and behavioral disorders. The new LDRT included conditions such as anxiety, depression, and suicide attempts as manifestations of work-related psychological stress. In the 1999 edition, depressive episodes, for example, were exclusively linked to exposure to toxic substances such as mercury and manganese.

This work has limitations, such as the possible underreporting of cases to be considered, given the often subjective nature of work-related mental and behavioral disorders and the lack of standardization and/or training of experts for the recognition of psychosocial risk factors at work. It is also necessary to consider that work injuries, categories in which work-related mental and behavioral disorders are situated, often underreported events in the Brazilian context, which may have resulted in an underestimation of the real incidence of these occurrences and distorted the presented data . Another element of imprecision to consider is the possible non-establishment of the causal nexus between work-related mental and behavioral disorders and work activity, either by the company, which may fail to issue the CAT, or by the medical expert, who may not recognize the NTP. Another relevant point is that the results cannot be generalized to informal workers, who do not have access to social security benefits covered by Social Security. The increasing informality in the Brazilian labor market may also have influenced this research results. Nevertheless, despite these considerations, this study data offer substantial contributions, addressing gaps not yet covered in recent research in the Workers’ Health field, notably regarding the temporal trend of work-related mental and behavioral disorders in Brazil, and provide evidence regarding the impact and effectiveness of NTP in the context of characterizing work-related mental and behavioral disorders.

## Conclusion

Before the implementation of the NTP (2003-2007), the average incidence of work-related mental and behavioral disorders was 15.59 per 100,000 RGPS links, with a growing trend (APC = 29.28%; p < 0.001), and more than doubled (43.77 per 100,000) after the implementation of this methodology (2008-2019). The trend of work-related mental and behavioral disorders after the changes implemented in 2007 in the recognition of the nexus between injury and work by the INSS (2008-2019) showed a decrease in the country (APC = -23.73%; p < 0.001), however, the rates doubled the average of the pre-NTP period, a pattern that was repeated for all administrative regions of the country.

These findings suggest that the implementation of a new system for establishing the NTP has changed the magnitude of the incidence of work-related mental and behavioral disorders in Brazil, indicating a possible improvement in the recognition and recording of these conditions by Social Security. This highlights the relevance and effectiveness of this methodological strategy as an auxiliary tool in characterizing the occupational nature of work-related mental and behavioral disorders within the scope of Social Security.

Finally, it emphasizes the importance of alignment between the observed increase in work-related mental and behavioral disorders trends and the response of the public authorities through strategies and public policies for comprehensive care and attention to workers. This includes those aimed at promoting and protecting physical and mental health, preventing illness due to psychosocial risk factors in the workplace, and addressing diseases related to these factors. Additionally, actions for vocational rehabilitation of those workers affected by such conditions are crucial.

## References

[1] World Health Organization (2022). World mental health report. Transforming mental health for all.

[2] GBD 2019 Mental Disorders Collaborators (2022). Global, regional, and national burden of 12 mental disorders in
204 countries and territories, 1990-2019: a systematic analysis for the
Global Burden of Disease Study 2019.. Lancet Psychiatry.

[3] Silva-Junior JS, Fischer FM (2014). Long-term sickness absence due to mental disorders is associated
with individual features and psychosocial work conditions. PLoS One.

[4] Ministério da Previdência Social Base de dados históricos da Previdência Social..

[5] Souza WF (2013). Transtornos mentais e comportamentais relacionados ao trabalho o
que a psicologia tem a dizer e a contribuir para a saúde de quem
trabalha?. Fractal Rev Psicol.

[6] Silva RAC (2011). A inclusão dos transtornos mentais como doença relacionada ao trabalho:
discursos sobre as dificuldades de reconhecimento dos nexos causais.

[7] Nicholson PJ (2018). Common mental disorders and work. Br Med Bull.

[8] Harvey SB, Modini M, Joyce S, Milligan-Saville JS, Tan L, Mykletun A (2017). Can work make you mentally ill A systematic meta-review of
work-related risk factors for common mental health problems. Occup Environ Med.

[9] Mikkelsen S, Coggon D, Andersen JH, Casey P, Flachs EM, Kolstad HA (2021). Are depressive disorders caused by psychosocial stressors at work
A systematic review with meta-analysis. Eur J Epidemiol.

[10] Van der Molen HF, Nieuwenhuijsen K, Frings-Dresen MH, Groene G (2020). Work-related psychosocial risk factors for stress-related mental
disorders: an updated systematic review and meta-analysis.. BMJ Open.

[11] Niedhammer I, Bertrais S, Witt K (2021). Psychosocial work exposures and health outcomes a meta-review of
72 literature reviews with meta-analysis. Scand J Work Environ Health.

[12] Oliveira PRA, Portela MC, Corrêa HR, Souza WR (2021). Nexo Técnico Epidemiológico Previdenciário (NTEP) risco das sete
atividades econômicas e condições incapacitantes mais frequentes, Brasil,
2000-2016. Cad Saúde Pública.

[13] Oliveira SG (2023). Indenizações por acidente do trabalho ou doença ocupacional..

[14] Instituto Nacional do Seguro Social Manual de acidente de trabalho..

[15] Pilegis OR (2019). Aferição do nexo causal nos transtornos mentais e comportamentais
relacionados ao trabalho por uma análise multiprofissional e
transdisciplinar do tema. Revista do Tribunal Regional do Trabalho da 15ª Região.

[16] Seligmann-Silva E, Bernardo MH, Maeno M, Kato M (2010). O mundo contemporâneo do trabalho e a saúde mental do
trabalhador. Rev Bras Saúde Ocup.

[17] Silva-Junior JS, Almeida FSS, Morrone LC (2012). Discussão dos impactos do nexo técnico epidemiológico
previdenciário. Rev Bras Med Trab.

[18] Bridi LRT (2023). Caminhos e descaminhos do Fator Acidentário de Prevenção (FAP): análise
histórica de uma política de saúde do trabalhador no Brasil.

[19] Antunes JLF, Waldman EA (2002). Trends and spatial distribution of deaths of children aged 12-60
months in São Paulo, Brazil, 1980-98. Bull World Health Organ.

[20] Antunes JLF, Cardoso MRA (2015). Uso da análise de séries temporais em estudos
epidemiológicos. Epidemiol Serv Saúde.

[21] Penfold RB, Zhang F (2013). Use of interrupted time series analysis in evaluating health care
quality improvements. Acad Pediatr.

[22] Turner SL, Karahalios A, Forbes AB, Taljaard M, Grimshaw JM, Cheng AC (2020). Design characteristics and statistical methods used in
interrupted time series studies evaluating public health interventions a
review. J Clin Epidemiol.

[23] Ewusie JE, Soobiah C, Blondal E, Beyene J, Thabane L, Hamid JS (2020). Methods, applications and challenges in the analysis of
interrupted time series data a scoping review. J Multidiscip Healthc.

[24] Brunoni AR, Suen PJC, Bacchi PS, Razza LB, Klein I, Dos Santos LA (2023). Prevalence and risk factors of psychiatric symptoms and diagnoses
before and during the COVID-19 pandemic findings from the ELSA-Brasil
COVID-19 mental health cohort. Psychol Med.

[25] Damiano RF, Caruso MJG, Cincoto AV, Rocca CCA, Serafim AP, Bacchi P (2022). Post-COVID-19 psychiatric and cognitive morbidity preliminary
findings from a Brazilian cohort study. Gen Hosp Psychiatry.

[26] World Health Organization (2017). Depression and other common mental disorders: global health
estimates.

[27] Silva-Junior JS, Razzouk D, Lima MAG, Cordeiro Q (2015). Saúde mental e trabalho.

[28] Wernke AR, Teixeira MCL, Kock BO, Sousa OLO, Melo ACMC, Sakae TM (2021). Taxas de risco de acidentes de trabalho no Brasil efeito do Fator
Acidentário de Prevenção (FAP)?. Ciênc Saúde Colet.

[29] Rodrigues AB, Santana VS (2019). Acidentes de trabalho fatais em Palmas, Tocantins, Brasil
oportunidades perdidas de informação. Rev Bras Saúde Ocup.

[30] Alves PFMT (2020). Psychiatric inconsistencies of technical epidemiological nexus
codes used by the Brazilian Social Service to classify work-related
disorders. Rev Bras Med Trab.

[31] Pessoa-Junior AR (2017). Crítica à sistemática do Nexo Técnico Epidemiológico Previdenciário e a
sua aplicação no direito do trabalho.

[32] Supremo Tribunal Federal (2020). Inteiro Teor de Acórdão - Ação Direta de Inconstitucionalidade 3.931,
Distrito Federal.

[33] Todeschini R, Codo W (2013). Uma revisão crítica da metodologia do Nexo Técnico Epidemiológico
Previdenciário (NTEP). Rev Baiana Saúde Pública.

[34] Lunardi MS, Soliman LAP, Pauli C, Lin K (2011). Epilepsy and workplace accidents in Brazil a national statistics
study. Arq Neuropsiquiatr.

[35] Costa DS (2016). Saúde do trabalhador: aplicação do nexo técnico epidemiológico
previdenciário à insuficiência venosa crônica.

[36] Teixeira EB (2011). Nexo técnico epidemiológico e os benefícios previdenciários por
acidentes de trabalho.

[37] Branco ABA, Ildefonso SAG (2012). Prevalence and duration of social security benefits allowed to
workers with asthma in Brazil in 2008. J Bras Pneumol.

[38] Almeida PCA, Barbosa-Branco A (2011). Acidentes de trabalho no Brasil prevalência, duração e despesa
previdenciária dos auxílios-doença. Rev Bras Saúde Ocup.

[39] Lombardi ALM (2017). O FAP e o NTEP como referenciais teóricos e práticos no estudo da
proteção e da prevenção acidentárias: aspectos trabalhistas e
previdenciários.

[40] Cardoso MCB, Araújo TM (2018). Atenção aos transtornos mentais relacionados ao trabalho nas
regiões do Brasil. Psicol Soc.

[41] Kawachi I (2024). The changing nature of work in the 21st century as a social
determinant of mental health. World Psychiatry.

[42] Silva-Junior JS, Fischer FM (2015). Afastamento do trabalho por transtornos mentais e estressores
psicossociais ocupacionais. Rev Bras Epidemiol.

[43] Ministério da Saúde (2023). Portaria nº 1.999, de 27 de novembro de 2023.. Diário Oficial da União.

